# Immunomodulation Through Fibroblast-Derived Extracellular Vesicles (EVs) Within 3D Polycaprolactone–Collagen Matrix

**DOI:** 10.3390/biomimetics10080484

**Published:** 2025-07-22

**Authors:** Afsara Tasnim, Diego Jacho, Agustin Rabino, Jose Benalcazar, Rafael Garcia-Mata, Yakov Lapitsky, Eda Yildirim-Ayan

**Affiliations:** 1 Department of Bioengineering, University of Toledo, Toledo, OH 43606, USA; afsara.tasnim@rockets.utoledo.edu (A.T.);; 2 Department of Biological Sciences, University of Toledo, Toledo, OH 43606, USA; 3 Department of Chemical Engineering, University of Toledo, Toledo, OH 43606, USA

**Keywords:** extracellular vesicles, macrophages, fibroblasts, polycaprolactone, collagen, inflammation, immunomodulation, tissue regeneration

## Abstract

Extracellular vesicles (EVs) have emerged as promising acellular tools for modulating immune responses for tissue engineering applications. This study explores the potential of human fibroblast-derived EVs delivered within a three-dimensional (3D) injectable scaffold composed of polycaprolactone (PCL) nanofibers and collagen (PNCOL) to reprogram macrophage behavior and support scaffold integrity under inflammatory conditions. EVs were successfully isolated from human fibroblasts using ultracentrifugation and characterized for purity, size distribution and surface markers (CD63 and CD9). Macrophage-laden PNCOL scaffolds were prepared under three conditions: macrophage-only (MP), fibroblast co-encapsulated (F-MP), and EV-encapsulated (EV-MP) groups. Structural integrity was assessed via scanning electron microscopy and Masson’s trichrome staining, while immunomodulatory effects were evaluated through metabolic assays, gene expression profiling, and immunohistochemistry for macrophage polarization markers (CD80, CD206). When co-encapsulated with pro-inflammatory (M1) macrophages in PNCOL scaffolds, fibroblast-derived EVs preserved scaffold structure and significantly enhanced macrophage metabolic activity compared to the control (MP) and other experimental group (F-MP). The gene expression and immunohistochemistry data demonstrated substantial upregulation of anti-inflammatory markers (*TGF-β*, *CD163*, and *CCL18*) and surface protein CD206, indicating a phenotypic shift toward M2-like macrophages for EV-encapsulated scaffolds relative to the other groups. The findings of this study demonstrate that fibroblast-derived EVs integrated into injectable PCL–collagen scaffolds offer a viable, cell-free approach to modulate inflammation, preserve scaffold structure, and support regenerative healing. This strategy holds significant promise for advancing immuno-instructive platforms in regenerative medicine, particularly in settings where conventional cell therapies face limitations in survival, cost, or safety.

## 1. Introduction

Extracellular vesicles (EVs) are lipid bilayer-enclosed particles secreted by virtually all cell types and are present in various biofluids, where they facilitate the transport of diverse bioactive molecules, including proteins, nucleic acids, lipids, and metabolites [1,2,3,4]. In recent years, EVs have gained significant attention due to their critical roles in autocrine, paracrine, and other biological signaling pathways. They mediate intercellular communication through receptor–ligand interactions and deliver functional cargo to recipient cells via receptor-mediated uptake or membrane fusion [5,6]. Proteomic analyses have shown that EVs are enriched with both coding and noncoding RNAs, as well as proteins involved in cell communication, membrane trafficking, and cytoskeletal dynamics [7]. These characteristics position EVs as promising candidates for various clinical applications, including pharmacological delivery agents, noninvasive biomarkers for early disease detection, treatment agents for metastatic diseases, and even for tissue regeneration [8].

In tissue regeneration applications, both cell-based (e.g., encapsulated or scaffold-seeded cells) and scaffold-based approaches are commonly employed to deliver therapeutic cells to the defect site while providing temporary structural support until *de novo* tissue is regenerated. Among these, mesenchymal stem cells (MSCs) are frequently utilized due to their multipotent differentiation potential and immunomodulatory properties [9]. However, using MSCs has its own challenges. For instance, the high number of cells for tissue regeneration requires time-demanding and costly in vitro expansion which can alter the functional properties of the stem cells [10]. Additionally, cell-based therapies often suffer from low survival rates of transplanted cells and uncontrolled differentiation at the target site, further limiting their efficacy. These limitations have led to growing interest in cell-free therapeutic strategies. Recent studies suggest that the paracrine factors secreted by MSCs, particularly EVs, can be explored as promising acellular agents for delivery within tissue scaffolds for tissue repair and immunomodulation [11].

Modulating the immune response is a critical factor in tissue regeneration. Among immune cells, macrophages have been extensively studied due to their pivotal role in interacting with both biomaterials and pathogens, thereby influencing the body’s immune response [12]. Macrophages are broadly categorized into two phenotypes: classically activated (M1) macrophages, which exhibit pro-inflammatory properties, and alternatively activated (M2) macrophages, which possess anti-inflammatory and tissue-repairing functions. The dynamic balance between these phenotypes is essential for orchestrating inflammation resolution, injury response, and tissue repair [12,13]. Recent studies have highlighted the importance of EVs in mediating communication between tissue cells and macrophages for resolving pro-inflammatory responses [14].

The MSC-derived EVs are widely studied for their immunomodulatory properties; however, MSC cultivation is time-intensive and costly in vitro, which can compromise their functional characteristics and therapeutic efficacy [15]. In contrast, fibroblasts are the most abundant stromal cells in connective tissue, playing a key role in extracellular matrix production, tissue remodeling, and cell–to-cell communication, making them a biologically relevant and scalable cell source [16]. Fibroblasts are relatively easy to isolate, highly proliferative under standard culture conditions, and more cost-effective to expand compared to MSCs, thus offering an efficient and sustainable EV source for translational applications [16]. Moreover, fibroblast-derived EVs have been shown to carry functionally relevant bioactive cargo—including extracellular matrix-associated proteins, signaling molecules, and immunomodulatory factors—comparable to EVs derived from other cell sources, reinforcing their potential in tissue repair and immunomodulation strategies [17].

While EVs have great potential for tissue regeneration and immunomodulation, their effective delivery to the defect site remains a critical challenge. Collagen-based scaffolds offer a promising solution, as collagen is the primary structural protein in connective tissues and a major component of the interstitial extracellular matrix (ECM) [18,19]. In addition, their injectability and low immunogenicity, compared to other natural or synthetic biomaterials, make them ideal candidates for immunomodulatory applications. However, a key limitation of collagen-based scaffolds is their rapid enzymatic degradation, which compromises mechanical stability and long-term structural support [20]. To address these issues, polycaprolactone (PCL), an FDA-approved, biocompatible, and biodegradable polymer, has been incorporated into collagen scaffolds to enhance structural integrity and slow degradation. This composite strategy leverages the mechanical strength and durability of PCL while retaining the bioactivity and biocompatibility of collagen [21,22].

To this end, the aim of this study is to explore the immunomodulatory potential of fibroblast-derived EVs delivered within a composite injectable three-dimensional (3D) scaffold composed of PCL nanofibers and collagen, called PNCOL. Specifically, we aimed (1) to characterize the physicochemical properties of EVs secreted by human dermal fibroblasts, and (2) to investigate whether fibroblast-derived EVs can modulate macrophage polarization toward an anti-inflammatory (M2-like) phenotype and concurrently preserve scaffold architecture within a 3D biomimetic matrix. Together, these aims address a significant gap in EV-based regenerative immunotherapy by integrating acellular signaling agents into a bioactive scaffold for targeted immune response modulation and structural preservation.

## 2. Materials and Method

### 2.1. Cell Cultivations and EV Isolation

**Fibroblast Cultivation as Extracellular Vesicles (EV) Producer Cells:** The human fibroblasts (HFF1, ATCC; USA) were used as EV producer cells. The fibroblasts were maintained in a complete medium of Dulbecco’s modified Eagle’s medium (DMEM, ATCC; USA) supplemented with 15% (*v*/*v*) heat-inactivated fetal bovine serum (FBS, Corning; USA), 1% penicillin-streptomycin and 1.5 g/L sodium bi-carbonate. The cultures were grown in T75 flasks at 37 °C and 5% CO_2_ until reaching approximately 70–80% confluency. The culture medium was then renewed by serum free-medium, specifically DMEM with 1% penicillin-streptomycin and 1.5 g/L sodium bi-carbonate, devoid of any fetal bovine serum (FBS), to eliminate pre-existing EVs that may already be present in the FBS.

**EV Isolation:** Following for another 48 h fibroblast culturing, the conditioned medium was collected for isolating EVs using a standard ultracentrifugation protocol that involved optimized sequential and differential centrifugation steps [23,24]. In brief, 30 mL of conditioned medium was obtained from around 3M of fibroblasts followed by centrifugation at 300× *g* for 10 min to remove cells. The resulting supernatant was centrifuged again at 2600× *g* for 10 min to exclude apoptotic bodies and larger cell debris. To eliminate micro-vesicles, the collected supernatant was then centrifuged at 10,000× *g* for 60 min. The centrifugation continued with the collected supernatant at a higher speed of 100,000× *g* for another 2 h. Following the removal of the supernatant, the remaining pellet, containing nano-scaled vesicles, was resuspended in 1 mL of 1X phosphate-buffered saline (PBS). The resuspended pellet underwent a subsequent centrifugation at 100,000× *g* for an additional 2 h to enhance the purity of the EVs. Finally, the collected EVs in pellets were resuspended as half in 1 mL of 1x PBS and the other half in 1 mL of lysis buffer. The lysis buffer was prepared by dissolving 1 tablet of PierceR Protease Inhibitor (Thermo Fisher Scientific, USA) in 10 mL of RIPA buffer (Sigma Aldrich, USA). All centrifugation steps were performed using a ‘Beckman Coulter J2-HC Centrifuge’ machine (USA) at 4 °C. The samples were consistently kept in ice to prevent further protein degradation from this point onwards. Beckman Coulter J2-HC Centrifuge’ machine (USA).

**Pro-inflammatory Macrophage Cultivation:** Prior incorporating pro-inflammatory macrophages with 3D scaffold, the human blood-derived U937 monocytes (U937, ATCC; Manassas, VA, USA) were cultivated and differentiated to naïve macrophages, followed by polarization into pro-inflammatory macrophages. Briefly, U937 monocytes were expanded in RPMI media supplemented with 10% (*v*/*v*) heat-inactivated fetal bovine serum (FBS; Corning; USA) and 1% penicillin/streptomycin. Upon reaching 80–90% confluency, differentiation into naïve macrophages was initiated by incubating these cells with 100ng/mL phorbol myristate acetate (PMA) (Sigma-Aldrich, St. Louis, MO, USA) for 24 h, followed by PMA withdrawal and resting for another 24 h. The macrophages underwent further differentiation into M1 macrophages after incubation with 100ng/mL lipopolysaccharide (LPS) (Sigma, Ronkonkoma, NY, USA) and 20 ng/mL IFN-γ (Peprotech, Cranbury, NJ, USA) for another 24 h based on our previously established protocol [25,26,27,28]. Then, upon confirming pro-inflammatory macrophage differentiation, the pro-inflammatory macrophages were encapsulated within all 3D Nanofibrous Scaffolds with 1 × 10^6^ cells/mL seeding density.

### 2.2. Synthesis of Macrophage-Laden 3D Nanofibrous Scaffold and Endogenous Loading of EV

**Figure 1:**
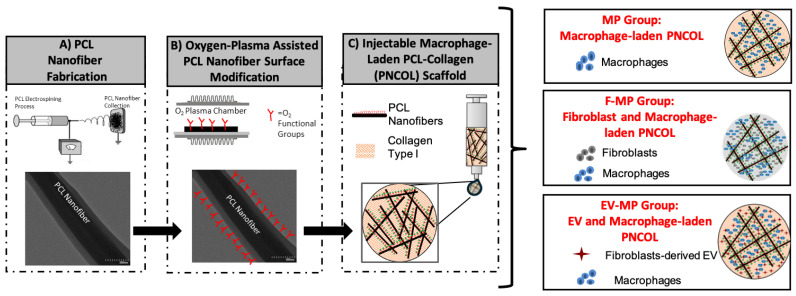
The schematic representations of major steps followed to create macrophage-laden 3D nanofibrous scaffold (MP) and MP with fibroblast cells (F-MP) and MP with fibroblast-derived EVs (EV-MP).

The nanofibrous macrophage-laden scaffold, called PNCOL, was fabricated based on our well-established protocols [22,29]. The process involved interspersing polycaprolactone (PCL) electrospun nanofibers within the macrophage encapsulated neutralized collagen type-I solution. The morphology and the injectability of the electrospun PCL nanofibers used in this study were also thoroughly characterized in our prior studies [21,22,29]. Figure 1 demonstrates the schematic representation of the macrophage-laden PNCOL scaffold synthesis and endogenous loading of EV.

Briefly, PCL (MW 45000, Sigma-Aldrich, USA) pellets were dissolved overnight inside a chemical fume hood in a mixture of chloroform and methanol solution at a 3:1 ratio, resulting in a final polymer concentration of 16% (*w*/*v*). The following day, the PCL solution was electrospun at a flow rate of 8 mL/h through a 20-gauge needle under 20 kV potential. Nanofibers were continuously collected for 2 h and then transferred under the chemical hood to allow for overnight residual solvent evaporation overnight. The next day, the PCL nanofibers were homogenized using a high-speed homogenizer (Ultra-Turrax, IKA Works, Inc.). To enhance hydrophilicity and improve cell affinity, the nanofibers were subjected to oxygen-plasma treatment for 3 min consistent with protocols from our prior studies [22,29]. In brief, the conjugated-PCL nanofibers were admixed with neutralized collagen type-I solution at 3% (*w*/*v*) concentration to prepare injectable PNCOL. The PCL nanofiber concentration (3%) was chosen based on their prior studies [21,29,30,31,32]. The PCL nanofibers were mixed within the neutralized collagen type-I solution with a 3% (*w*/*v*) concentration along with macrophages with 1 × 10^6^ cells/mL seeding density. The neutralized collagen type-I solution with 3 mg/mL concentration was prepared from 9.1 mg/mL collagen type-I stock solution (Rat Tail Collagen, Purity = 90%, Corning, USA) with a pH of ~3.4 using 1M NaOH, PBS, and deionized water.

The macrophage-laden 3D Nanofibrous scaffold, ***named MP***, served *as a control* for this study. To understand the role of immunomodulation capacity of fibroblast cells and fibroblast-derived EVs, two additional experimental groups were also used. One of the experimental groups was ***called F-MP*** in which fibroblast cells were incorporated within the macrophage-laden 3D Nanofibrous scaffold. The other experimental group was ***called EV-MP*** in which EVs were incorporated within the macrophage-laden 3D Nanofibrous scaffolds with a concentration of 0.2 µg/µL. From each group, 300 µL volume of scaffolds were prepared for validation, structural, and cellular characterizations. During the study period (5 days), the control group (MP) was cultured in complete RPMI media, while F-MP and EV-MP groups were cultured in 50:50 mixture of complete RPMI and DMEM media. In every group, the media was replaced with 50% fresh media every two days.

### 2.3. Confirmation and Characterization of Fibroblast-Derived Extracellular Vesicles

To confirm the presence of extracellular vesicles (EVs), the biological, hydrodynamic, and structural characterizations were conducted using antibody staining, nanoparticle size distribution analysis, and scanning electron microscopy (SEM), respectively.

***EV specific membrane protein marker analysis:*** To confirm the successful EV isolation, EV-specific membrane protein markers CD63 (10628D, Invitrogen, USA) and CD9 (10626D, Invitrogen, USA) were identified using Western blotting analysis. β-actin (MA5-15739-HRP, Invitrogen, USA) was used as a loading control since it is expressed by all eukaryotic cell type and does not fluctuate significantly in response to different cellular treatments, which further supports its suitability as a control protein [33]. Briefly, following isolating the fibroblast-derived EVs using ultracentrifugation (Section 2.1), they were lysed and sonicated, followed by centrifugation for 10 min at 4 °C. Then, the proteins were separated by Sodium Dodecyl-Sulfate Polyacrylamide Gel Electrophoresis (SDS-PAGE) with a 13% resolving gel and 5% stacking gel, and then transferred to a 0.22 µm polyvinylidene fluoride membrane (Millipore) for immunoblotting with mouse beta-Actin monoclonal antibody (BA3R, ThermoFisher USA), mouse exosome-anti-CD9 (TS9, ThermoFisher USA), and mouse exosome-anti-CD63 (TS63, ThermoFisher USA), respectively, diluted in blocking solution (1:2000). The sodium azide was added (1:1000) to preserve the antibodies. Bands were visualized by Goat anti-mouse IgG1 horseradish peroxidase conjugate diluted in blocking solution (1:20,000), and immobilon western chemiluminescent HRP Substrate ‘Supersignal WEST Pico Plus’—peroxide and luminol mixed freshly at a ratio of 1:1. The cassette was then taken to the dark room, where an autoradiography film (Santa Cruz, SC201697, ThermoFisher USA) was placed on top of the membrane blot. The film was exposed for a desired time and then developed in an X-ray machine, CCD camera, or chemiluminescent imaging system to capture the protein bands.

***Particle Size Distribution Analysis:*** Following biological confirmation of EVs using EV-specific surface proteins, the size distribution of particles within the sample was also assessed. The analysis was performed using dynamic light scattering (DLS) at a wavelength of 633 nm and a detector angle of 173°, with a Zetasizer Nano ZS (Malvern, UK) instrument that usually combines both the dynamic and the electrophoretic light scattering techniques. Additional measurements were performed at a detector angle of 13° to verify that the results were independent of the detector angle used. The z-average nanoparticle diameter and size dispersity were obtained by analyzing the intensity autocorrection functions through cumulant analyses. For all DLS measurements, isolated EVs were diluted in 1× PBS, vortexed for 5 seconds, and placed in disposable polystyrene Fisherbrand cuvettes, where each measurement was taken twice.

***Visualization of Fibroblast-derived EVs:*** The EVs were visualized using Scanning Electron Microscopy (SEM) (Hitachi, USA). Briefly, the samples were first fixed using a 4% paraformaldehyde in PBS solution for 30 min. Following fixation, the samples underwent a dehydration process in sequential ethanol solution with increasing concentrations, ranging from 30% to 100% for 15 min each. The samples were then successfully exposed for 10 min to ethanol/hexamethyldisilane (HMDS) solutions ranging from 30% to 100% to continue the dehydration process to enhance the image quality. The samples were air-dried overnight, followed by gold-sputter coating to allow for visualization under SEM.

### 2.4. Structural Changes in Macrophage-Laden 3D Nanofibrous Scaffold upon EV Incorporation

To assess structural changes in fibrous structure and density of macrophage-laden 3D Nanofibrous Scaffold (MP) following fibroblast incorporation (F-MP) and fibroblast-derived EV incorporation (EV-MP), Masson’s trichrome and scanning electron microscopy (SEM) and quantitative image analysis were performed. For Masson’s trichrome staining, on characterization day, the scaffolds were fixed in 10% formalin overnight, dehydrated in graded ethanol, and embedded in paraffin. Thin sections of tissue with a thickness of 5 μm were obtained using a microtome (GMI-Reichert Jung 820 II) and mounted on a glass microscopic slide (Mercedes Medical MER 7200/45/BL). The sections were then stained with Masson’s trichrome to visualize collagen fiber distribution within the scaffold. For SEM images, the same SEM protocol in Section 2.3 was followed. The SEM images were further used to understand the changes in porosity. For image analysis, the ImageJ (NIH, USA) were used to distinguish pore regions from fibrous regions based on pixel intensity. A uniform threshold range was applied across all samples to ensure comparability. Following thresholding images were binarized, with pores exhibiting red regions and the fibrous matrix as gray. The total pore area and overall image area were determined, and percent porosity was calculated by dividing the pore area by the total area.

### 2.5. Cellular Metabolic Activity and Total Protein Concentration in Each Group of Scaffold

The cellular metabolic activity of each group of scaffolds was assessed via colorimetric-based CyQUANT XTT cell viability assay (ThermoFisher Scientific, Waltham, MA, USA) measuring the amount of formazan red as converted by the cellular secretions, thereby measuring cell metabolism. Briefly, the media from each group of scaffolds was collected in triplicates at days 1, 3, and 5 and mixed with XTT reagent in a 100:70 µL (media: XTT) ratio in a 96-well plate. After 3 h of incubation, absorbance reading was taken at 450 and 660 nm using a UV kinetic microplate reader. The complete RPMI media absorbance reading was used as a blank for control group (MP), while a mixture RPMI and complete DMEM media (50:50) was used as the blank for the experimental groups (F-MP and EV-MP). After subtracting 660 nm reading from the 450 nm and blank absorbance reading, the data was normalized with Day 1 values for each group to assess the changes in cellular metabolic activity over time.

### 2.6. Phenotypic Changes of Macrophages Within 3D Scaffolds

**Gene Expression Analysis:** Quantitative real-time polymerase chain reaction (RT-qPCR) was performed to evaluate the expression levels of both pro-inflammatory and anti-inflammatory genes (Table 1) to understand the influence of EV treatment on macrophage polarization dynamics. Briefly, RNA was isolated from the scaffolds using TRIzol reagent (ThermoFisher, USA). Prior to reverse-transcriptase, the scaffolds were mechanically crushed and smashed. Following the manufacturer’s instructions, the isolated RNA was then reverse transcribed into cDNA using Dircet-zolTM RNA Miniprep Plus kit (Zymo Research, USA) was used for the RT-PCR, which was carried out on the iCycler iQ detection system (Bio-Rad, USA). GAPDH was used as the housekeeping normalizing gene, where ∆∆Ct method was followed for obtaining the relative gene expression to determine the fold difference in gene expression between strained samples and non-loaded control samples. The primer sequences for each gene were designed using NBCI primer blast software and synthesized by integrated DNA Technologies (IDT), USA. Following is the table (Table 1) containing the list of genes and related primer sequences that are assessed in this study.

**Cell Surface Markers Analysis (Immunohistochemistry):** The macrophage-laden PCL–collagen matrices in all experimental groups (F-MP and EV-MP) and control group (MP) were fixed in 10% formalin, dehydrated through an ethanol gradient, and embedded in paraffin. Tissue matrices were sectioned at a thickness of 40 μm using a microtome (GMI-Reichert Jung 820 II), and mounted on a glass microscopic slide (Mercedes Medical MER 7200/45/BL), followed by overnight incubation at 80 °C. The next day, residual paraffin was removed from the slides by immersing them in xylene for 2 h and subsequent ethanol washes at 100%, 95%, 70%, and 50% concentrations followed by a final rinsing in diH2O for rehydration. Antigen retrieval was initiated with enzymatic treatment at 37 °C for 30 min by incubating the slides in a solution of trypsin and calcium chloride (0.1% (*v*/*v*) trypsin from ATCC, USA, and 0.1% (*w*/*v*) calcium chloride from Sigma-Aldrich, USA) Heat-mediated antigen retrieval was then performed at 95 °C for 30 min by immersing the slides in citrate buffer (10 mM sodium citrate in 0.05% Tween 20). Following permeabilization with Triton X-100 for 10 min, samples were washed in PBS and then blocked with 2.5% (*v*/*v*) goat serum (Invitrogen) and 0.2% Tween for 20 min to prevent non-specific binding. Overnight incubation at 4 °C was conducted with mouse monoclonal CD80 antibody (1:100, Invitrogen, TA501575) and recombinant rabbit CD206 antibody (1:100, ThermoFisher, MA1-35936), selected as pro-inflammatory and anti-inflammatory macrophage-related surface markers, respectively. After washing in PBS and 0.2% Tween solution, slides were re-blocked in the goat serum solution for 20 min at room temperature. Secondary staining involved a 4.5 h incubation with Alexa Fluor 488 Phalloidin (1:200, ThermoFisher, A12379), goat anti-rabbit IgG (H+L) Highly Cross-Adsorbed Secondary Antibody Alexa Fluor 594 (1:200, ThermoFisher, A32740), and goat anti-mouse IgG (H+L) Highly Cross-Adsorbed Secondary Antibody, Alexa Fluor Plus 800 (1:200, ThermoFisher, A32730). Finally, nuclear counterstaining was performed with DAPI (4′,6-Diamidino-2-Phenylindole) dye (1:1000, Life Technologies) in PBS for 30 min, followed by a 0.2% Tween washing.

**Confocal imaging:** Confocal microscopy was conducted using a 63x oil or 20x dry objective with a Leica Stellaris 5 confocal system equipped with HyD detectors and the LASX software. Imaging was performed across at least three independent experimental replicates to visualize nuclei, F-actin cytoskeleton, CD80, and CD206 markers.

**Fluorescence Image Quantification:** Immunofluorescence images for CD80 (M1 marker) and CD206 (M2 marker) expression were analyzed using ImageJ software. Individual color channels were separated, and regions of interest (ROIs) containing cells were defined by thresholding. Mean fluorescence intensity for each marker was quantified within the ROIs, and data were normalized to the number of nuclei (DAPI staining) to account for variations in cell density.

### 2.7. Statistical Analysis

For this study, n = 15 biological replicates per group were used, with each biological replicate representing an independently prepared scaffold. All quantitative measurements were performed in triplicate (3 technical replicates per sample) to account for assay variability and ensure reproducibility. Except for Western blot analysis, where biological characterization of each group of scaffolds was performed using EV-specific protein markers CD9 and CD63, with β-actin serving as a loading control. For this, two independent scaffold samples from each group were processed by adding them to 1 mL of lysis buffer, followed by thorough homogenization using vortex mixing to ensure proper protein extraction.

RStudio was used for statistical analysis, where all the data were represented as the mean value plus/minus the standard deviation. As we have three different groups in our study, a one-way ANOVA with a post-hoc Tukey test was used to determine the differences between groups. (*) indicates the statistical difference between MP group (control) and experimental groups with a significance level of *p* < 0.05. (**) indicates the statistical difference between MP group (control) and experimental groups with a significance level of *p* < 0.005. (***) indicates the statistical difference between MP group (control) and experimental groups with a significance level of *p* < 0.0001. (#) shows the statistical difference experimental groups (F-MP and EV-MP) with a significance level of *p* < 0.05. (##) shows the statistical difference experimental groups (F-MP and EV-MP) with a significance level of *p* < 0.005. (###) indicates the statistical difference between MP group (control) and experimental groups with a significance level of *p* < 0.0001.

## 3. Results

### 3.1. Human Fibroblast Secrete EVs with Distinct Surface Markers and Proper Sizes

**Figure 2:**
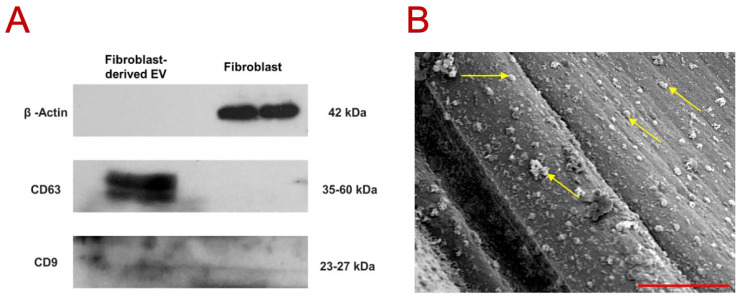
Characterization of fibroblast-derived EVs. (**A**) Western blot analysis of the EV-specific surface markers CD63 and CD9, β-actin as control where two independent scaffold samples per group were processed for Western blotting. (**B**) The representative image of fibroblast-derived EVs under SEM. The yellow arrows point the representative EVs. The scale bar is 3 µm.

The Western blot results (Figure 2A) confirmed the prominent presence of EV-specific membrane markers CD63 and CD9 in the EV sample, in contrast to the fibroblast-containing sample. Notably, β-actin (~42 kDa) was detected exclusively in the fibroblast sample, further validating the purity and identity of the isolated EVs. CD63 was strongly expressed in the EV fraction, consistent with previous reports indicating a characteristic band size between 30 and 60 kDa [36,37]. In comparison, CD9 exhibited a weaker and more diffuse band within the 23–27 kDa range, which aligns with MISEV2023 report indicating variable expression patterns of tetraspanin markers depending on EV source, cell type, and isolation methods [38]. Nevertheless, studies on fibroblast-derived EVs have consistently demonstrated detectable levels of both CD63 and CD9 [17,39].

The DLS analysis of the particle size indicated a z-average diameter of 226 ± 17 nm and a moderate size dispersity index of 0.214 ± 0.033 (mean ± SD). Given that larger particles scatter light much more intensely than smaller ones [40,41], these DLS point to most particles likely fall below ~200 nm in diameter, with the larger particles (despite their substantial contribution to the overall scattering signal) being relatively few in number. Likewise, the modest size dispersity index suggests limited aggregation or contamination by larger vesicular structures or cellular debris. This distribution aligns well with previous reports that characterize EV diameters generally within the 50–200 nm range [42,43]. Specifically, fibroblast-derived EVs are known to range from 30 to 150 [17,44], and one study reports a mean size of 180 ± 5 nm [45], which is consistent with the current dataset. Overall, this DLS analysis supports the conclusion that the isolated vesicles predominantly fall within the expected size range for EVs, indicating a high-quality isolation process with minimal contamination. The representative scanning electron microscopy (SEM) image (Figure 2B) presents the small dot-like features (pointed out with yellow arrow), distributed uniformly and prominently over the fibers, consistent in size and shape with extracellular vesicles (EVs). Considering the scale bar in SEM image (3 µm), these dots fall within the nanoscale range, reinforcing their compatibility with the EV size profile established through both literature [17,42,43,44,45] and the above DLS results. Furthermore, the absence of large aggregates indicates a well-purified EV preparation process.

### 3.2. The Effect of Fibroblast-Derived EVs on Structure of Encapsulated 3D Nanofibrous Scaffold

The structural changes of macrophage-laden 3D nanofibrous scaffold with and without EV incorporation and with fibroblast encapsulation were studied by assessing the structural integrity of collagen matrix in different scales using histological analysis (Masson’s trichrome) and in SEM imaging.

**Figure 3:**
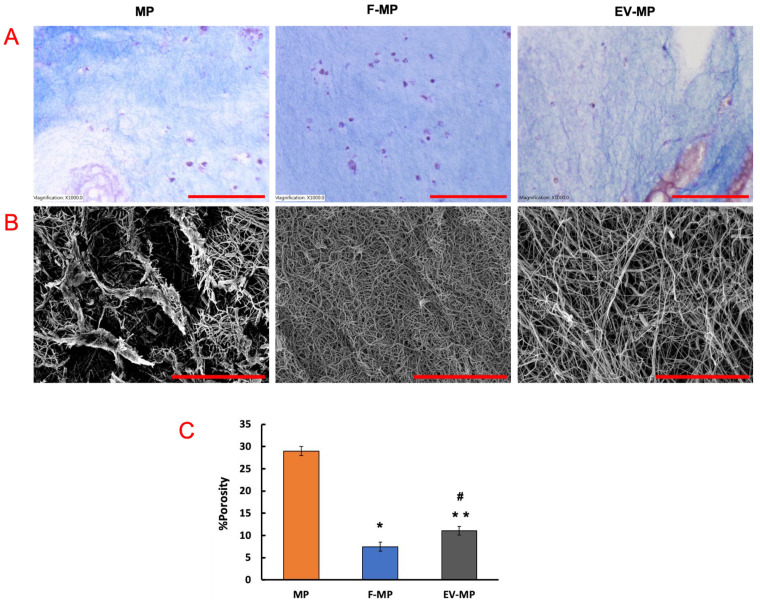
For 3D nanofibrous scaffold encapsulated with macrophages (MP), with macrophages and fibroblast (F-MP) and with macrophages and EV (EV-MP), n = 3 technical replicates per sample were used. (**A**) Masson’s trichrome staining histological images. The scale bar is 100 μm with 1000× magnification. (**B**) The SEM images. The scale bar is 10 μm and (**C**) The changes in percentage porosity in each group. (*) indicates the statistical difference between MP group (control) and experimental groups with a significance level of *p* < 0.05. (**) indicates the statistical difference between MP group (control) and experimental groups with a significance level of *p* < 0.005. (#) shows the statistical difference experimental groups (F-MP and EV-MP) with a significance level of *p* < 0.05.

While Masson’s trichrome staining (Figure 3A) stains collagen fibers blue and cell nuclei purple, providing macroscale information about the scaffold, the SEM images (Figure 3B) offer complementary nanoscale insight into fiber structure. In Figure 3A, the MP group showed relatively sparse collagen content throughout the scaffold. As expected, the F-MP group demonstrated enhanced collagen intensity and distribution, consistent with extracellular matrix (ECM) production of fibroblast. Interestingly, the EV-MP group exhibited a relatively dense collagen matrix compared to MP group, suggesting that extracellular vesicles contributed to regulating ECM structure. For SEM images (Figure 3B), in MP group, collagen fibers appeared sparsely arranged with minimal inter-fibrillar connectivity while the F-MP group exhibited a densely compacted fibrillar network, indicative of robust ECM production by fibroblasts. The EV-MP group revealed an intermediate but coherent fiber morphology with reduced sparsely formed fibers, suggesting that EVs play a role in modulating fiber integration at the nanoscale. The quantitative analysis of porosity confirmed the results from the histological and SEM images. The porosity was higher in MP group with ~30% value compared to F-MP and EV-MP groups. This aligns with qualitative observations of an enhanced collagen network, with matrix remodeling and deposition by fibroblasts (Figure 3B). The EV-MP group has a porosity of ~11% which is significantly lower than the MP group (*p* < 0.005), and slightly higher than the F-MP group (*p* < 0.05). This trend is consistent with the Masson’s trichrome (Figure 3A) and SEM data (Figure 3B), demonstrating a moderate matrix architecture—coherent and better integrated than MP alone, yet not as densely compacted as F-MP. Together, these findings highlight the role of EVs in preserving scaffold structure, mitigating the matrix-degrading effects of pro-inflammatory macrophages, and supporting a more stable tissue microenvironment.

### 3.3. EVs Increased Cellular Metabolic Activity Within M1-Macrophage Laden PNCOL Scaffold

Following the observation of notable structural differences among the scaffold groups, particularly between those with and without EVs, next a comprehensive biological analysis was conducted to understand whether observed matrix remodeling was driven by the differences in cellular activity including metabolic activity and phenotypic shifts in macrophages.

**Figure 4:**
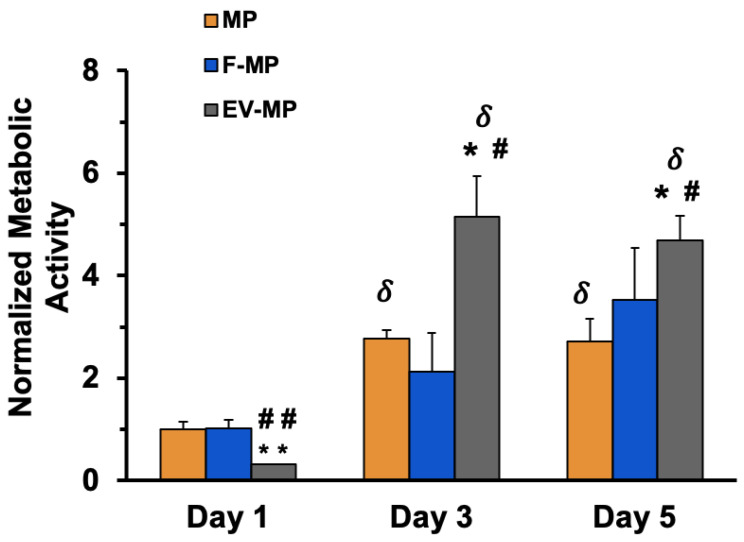
The changes in normalized metabolic activity according to Day1 over a 5-day period, n = 3 technical replicates per sample were used. (*) indicates the statistical difference between MP group (control) and experimental groups with a significance level of *p* < 0.05. (**) indicates the statistical difference between MP group (control) and experimental groups with a significance level of *p* < 0.005. (#) shows the statistical difference experimental groups (F-MP and EV-MP) with a significance level of *p* < 0.05. (##) shows the statistical difference experimental groups (F-MP and EV-MP) with a significance level of *p* < 0.005. (δ) indicates the statistical difference compared to Day1 within the same experimental group with a significance level of *p* < 0.05.

Figure 4 illustrates the temporal changes in cellular metabolic activity of encapsulated within PNCOL scaffolds under three experimental conditions: MP, F-MP, and EV-MP. The XTT assay readings were normalized to Day 1 within each group to evaluate metabolic shifts over time. On Day 1, all groups exhibited low metabolic activity, with no statistically significant difference between MP and F-MP group. Only the EV-MP group exhibited significantly lower metabolic activity compared to both MP and F-MP groups (*p* < 0.005) on Day 1. On both Day 3 and Day 5, a significant increase in metabolic activity was observed in the EV-MP group compared to both MP and F-MP groups on the same day (*p* < 0.05). This elevated metabolic activity remained significantly higher (~5-fold increase compared to Day 1) on Day 5 in the EV-MP group. The MP group also showed a modest but statistically significant increase starting from Day 1 until Day 5 (*p* < 0.05), while the F-MP group did not exhibit any significant changes. These findings indicate that the presence of EVs substantially enhances the metabolic activity of macrophages over time, potentially through mechanisms involving phenotypic changes. However, further investigations at molecular level are needed to elucidate the precise underlying mechanisms in this specific context.

### 3.4. EVs Modulates Macrophage Phenotype Towards Anti-Inflammatory Within 3D Matrix

**Figure 5:**
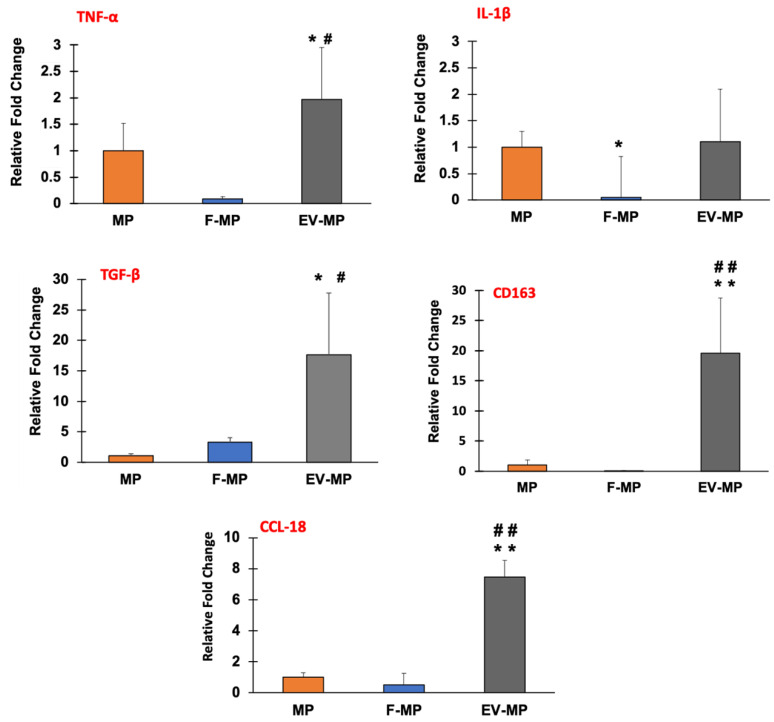
The changes in relative gene expression of pro-inflammatory (TNF-α and IL-1β) and anti-inflammatory genes (TGF-β, CD163, and CCL-18) for MP, F-MP, and EV-MP groups.), n = 3 technical replicates per sample were used. (*) indicates the statistical difference between MP group (control) and experimental groups with a significance level of *p* < 0.05. (**) indicates the statistical difference between MP group (control) and experimental groups with a significance level of *p* < 0.005. (#) shows the statistical difference experimental groups (F-MP and EV-MP) with a significance level of *p* < 0.05. (##) shows the statistical difference experimental groups (F-MP and EV-MP) with a significance level of *p* < 0.005.

The phenotypic changes of macrophages for MP group (within 3D PCL–collagen matrix), F-MP (within 3D PCL–collagen matrix along with fibroblast cells), and EV-MP group (within 3D PCL–collagen matrix along with fibroblast-derived EV) were assessed using pro- and anti-inflammatory gene expression analysis and confirmed via surface protein markers through immunohistochemistry (IHC). Figure 5 demonstrates the gene expression data providing relative fold changes of pro-inflammatory genes TNF-α and IL-1β along with the anti-inflammatory genes TGF-β, CD163, and CCL-18 for MP (control), F-MP, and EV-MP groups.

Regarding the pro-inflammatory markers, while there was a subtle yet statistically significant (*p* < 0.05) increase in TNF-α expression in the EV-MP group compared to both MP and F-MP groups, there was no statistically significant difference between MP and F-MP groups. For IL-1β expression, no statistical difference was observed between MP and EV-MP groups. However, IL-1β expression was slightly decreased with statistical significance (*p* < 0.05) in the F-MP group compared to MP. In contrast, anti-inflammatory gene expressions demonstrated a robust and consistent upregulation, especially in the EV-MP group. TGF-β expression increased dramatically to nearly 20-fold in EV-MP, which was significantly higher than both MP (around 2-fold) and F-MP (around 4-fold) groups. A similar trend was observed in the expressions of another key anti-inflammatory markers, CD163 and CCL-18. Both MP and F-MP groups exhibited low CD163 and CCL-18 expression levels, with no statistically significant difference between them. In contrast, the EV-MP group demonstrated a robust upregulation around 20-fold for CD163 and around 7-fold for CCL18, which were highly significant (*p* < 0.005) compared to both MP and F-MP groups. This suggests that fibroblast-derived EVs strongly enhance anti-inflammatory gene expressions of macrophages when cultured together within 3D PCL–collagen and further promote anti-inflammatory macrophage phenotype.

**Figure 6:**
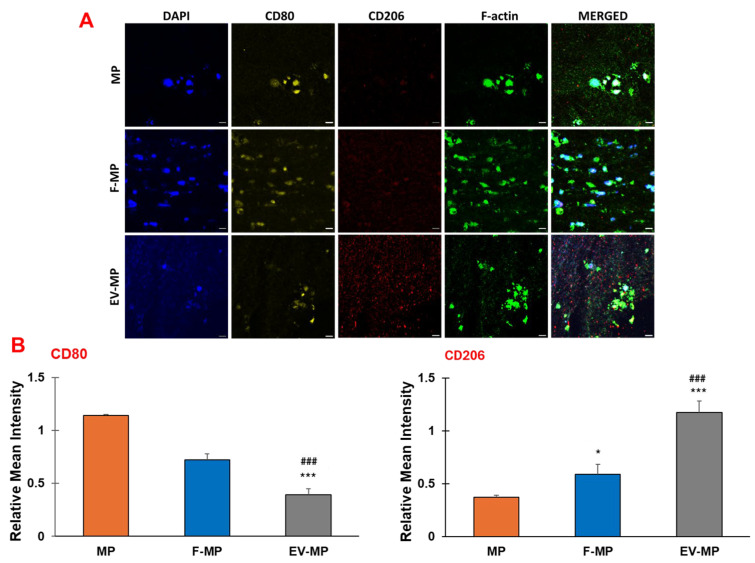
(**A**) Confocal microscopy images of the cell nucleus stained with DAPI, F-actin with phalloidin, pro-inflammatory (CD80), and anti-inflammatory marker (CD206). Representative of 5 images per group. Scale bar = 10 μm. (**B**) Quantitative fluorescence intensity analysis of pro-inflammatory marker (CD80) and anti-inflammatory marker (CD206) across groups. Data are presented as mean ± SD; n = 3 technical replicates per group. (*) indicates the statistical difference between MP group (control) and experimental groups with a significance level of *p* < 0.05. (***) indicates the statistical difference between MP group (control) and experimental groups with a significance level of *p* < 0.0001. (###) indicates the statistical difference between MP group (control) and experimental groups with a significance level of *p* < 0.0001.

To verify the findings of the gene expression analysis, the presence of pro- and anti-inflammatory surface proteins CD80 and CD206, respectively, was visualized using confocal microscopy. In Figure 6, the pro-and anti-inflammatory and surface markers along with cell nuclei and filamentous actin (F-actin) were demonstrated for MP, F-MP, and EV-MP groups. The cell nuclei and filamentous actin (F-actin) were tagged with DAPI and phalloidin, respectively. The pro-inflammatory (CD80) and anti-inflammatory (CD206) surface protein presence were assessed using CD80, and CD206 antibodies, respectively.

Aligned with the gene expression results, IHC confocal images demonstrated phenotypic shift in macrophages into anti-inflammatory phenotype in fibroblast-derived EV group (EV-MP) compared to control (MP) and F-MP group. For pro-inflammatory macrophage group (MP) within the 3D matrix, a strong presence of CD80 (red, pro-inflammatory) with minimal CD206 (green, anti-inflammatory) expression was observed, confirming a predominantly pro-inflammatory phenotype of macrophages. The co-culture with fibroblasts (F-MP group) slightly altered the macrophage phenotype. While CD80 signal remained apparent, CD206 expression increased modestly. As consistent with gene expression data, a dramatic shift toward an anti-inflammatory phenotype was visualized when pro-inflammatory macrophages cultured with fibroblast-derived EV within 3D matrix (EV-MP group). CD206 staining was notably intense and widespread, while CD80 was substantially reduced, indicating polarization of macrophages towards anti-inflammatory phenotype.

Quantification of immunofluorescence confirmed the immunomodulatory effects of fibroblast-derived EVs within the PNCOL scaffold (Figure 6B). CD80 expression was significantly reduced in the EV-MP group (0.40 ± 0.05) compared to both MP (1.14 ± 0.02; *p* < 0.001) and F-MP groups (0.72 ± 0.05; *p* < 0.05). Conversely, CD206 expression increased significantly in the EV-MP group (1.17 ± 0.1) relative to MP (0.37 ± 0.02; *p* < 0.001) and F-MP groups (0.59 ± 0.09; *p* < 0.01). These results demonstrate that EV incorporation promotes M2-like macrophage polarization while suppressing pro-inflammatory phenotypes within the scaffold.

## 4. Discussion

The immune microenvironment plays a pivotal role in determining the success of tissue-engineered constructs, particularly in inflammatory conditions where immune cell behavior can either support or disrupt regeneration [46,47]. Among immune modulators, in recent years, extracellular vesicles (EVs) have gained attention for their ability to modulate macrophage phenotypes through the delivery of bioactive cargo [48,49,50]. While EVs derived from stem cells have been widely studied, the immunomodulatory potential of fibroblast-derived EVs remains unexplored. In addition, fibroblasts were selected as the EV source in this study, due to their abundance as stromal cells in connective tissues, and their crucial roles in intracellular signaling, tissue remodeling, and extracellular matrix production [15]. Compared to mesenchymal stem cells (MSCs), fibroblasts offer an efficient, scalable, and more cost-effective platform for large-scale EV production under standard culture condition [15,16]. Furthermore, fibroblast-derived EVs have been shown to carry a rich cargo of extracellular matrix-associated proteins, signaling molecules, and immunoregulatory factors comparable to EVs derived from other cell types, supporting their relevance for tissue regeneration and immunomodulatory applications [17]. In this study, we aimed to investigate whether fibroblast-derived EVs could reprogram macrophage polarization toward an anti-inflammatory (M2-like) phenotype within 3D PCL–collagen matrix that mimics extracellular matrix conditions. Prior to investigating the immunomodulatory role of EVs, it was crucial to prove that EVs were derived from fibroblast. The molecular characterization (Western blot) (Figure 2) of extracted EVs confirmed the successful isolation of EVs secreted by human fibroblasts.

Following the successful validation of fibroblast-derived EVs, their functional impact was next assessed within the 3D PCL–collagen matrix, beginning with an evaluation of how EVs influence the structural integrity of the scaffold in the presence of pro-inflammatory macrophages. Together, the microscale (histological-Figure 3A) and nanoscale (SEM-Figure 3B) analyses offer crucial information for evaluating the structural integrity of the macrophage encapsulated scaffold across multiple spatial resolutions. Figure 3 highlights the critical role of EVs in preserving scaffold structure under pro-inflammatory conditions. In this study, the encapsulated macrophages were pro-inflammatory phenotype (M1), which are known to contribute to matrix degradation through the release of inflammatory cytokines and matrix metalloproteinases [25,51]. This effect was evident in the MP group, where reduced collagen integrity and sparsely distributed fibers were observed. However, in the EV-MP group, the presence of EVs mitigated these effects, as demonstrated by a more continuous and compact collagen fiber morphology both at the macroscale (Masson’s trichrome staining) and nanoscale (SEM imaging). Preserving the structural integrity of the scaffold is essential for effective immunomodulation, particularly in highly inflammatory environments. A stable extracellular matrix not only supports mechanical function but also serves as a critical microenvironment for modulating immune cell behavior. By maintaining the scaffold’s physical framework, EVs help stabilize the delivery system and protect it from inflammatory degradation, thereby enhancing their therapeutic potential in regulating immune responses.

Beyond contributing to structural integrity of the encapsulated matrix, the fibroblast-derived EVs further enhance the metabolic activity of macrophages when they encapsulated together within the 3D matrix and promote the macrophage polarization towards anti-inflammatory phenotype. The increased metabolic activity observed in macrophages co-cultured with fibroblast-derived EVs (Figure 4) which can be attributed to the direct physical interaction of EVs and macrophages and uptake of EVs by macrophages, which has been shown in the literature to significantly influence macrophage activation state and functional metabolism [52,53]. The 3D scaffold microenvironment may facilitate close spatial proximity and sustain interactions between EVs and macrophages, enhancing the likelihood of vesicle uptake and content delivery. The EV update process has been linked to increased mitochondrial activity and ATP production as macrophages shift toward more active functional states [54]. Both scaffold structural characterization data (Figure 3) and metabolic activity data (Figure 4) together suggest that fibroblast-derived EVs do not merely act as passive carriers of molecular signals but serve as active physical entities within the 3D matrix capable of modulating macrophage metabolism through direct cell-vesicle interactions. This interaction-driven metabolic shift is particularly relevant in tissue engineering contexts where controlling immune cell activity is critical to scaffold integration and regeneration. Thus, next, the phenotypic changes of macrophages were assessed within the 3D matrix with direct co-culturing with fibroblasts (F-MP group) and direct co-culturing with EVs (EV-MP) group.

The gene expression (Figure 5) and immunohistochemistry (Figure 6) data indicated a clear phenotypic shift toward M2-like macrophage polarization in EV-MP group. The TGF-β expression increased nearly 20-fold, and CD163 and CCL18 were upregulated by ~20-fold and ~7-fold, respectively (Figure 5) which far exceeded counterparts in both the MP and F-MP groups. The immunohistochemistry results (Figure 6A,B) visually confirmed the transcriptional data, demonstrating that fibroblast-derived EVs not only suppress pro-inflammatory features but robustly promote the expression of anti-inflammatory surface protein (CD 206), consistent with an M2-like macrophage phenotype. The quantitative immunofluorescence analysis further corroborates the immunomodulatory potential of fibroblast-derived EVs within the PNCOL scaffold. A significant reduction in CD80 expression, coupled with increased CD206 expression, indicates a shift toward an anti-inflammatory, M2-like macrophage phenotype in the EV-MP group. These results are consistent with the immunohistochemistry observations, collectively demonstrating that EV incorporation effectively promotes the anti-inflammatory differentiation of M1 macrophages conducive to tissue repair and scaffold stability under inflammatory conditions. The phenotypic changes can be explained with the physical interaction between fibroblast-derived EVs and macrophage. EVs engage with macrophages through membrane fusion, endocytosis, or surface adhesion, processes that alter macrophage membrane tension and cytoskeletal dynamics, and endosomal trafficking [55,56]. Within the 3D PCL–collagen scaffold, such physical interaction might have been enhanced, providing spatial and mechanical cues that are absent in traditional 2D culture systems. Unlike the F-MP group, which showed only a modest ~4-fold increase in TGF-β and no significant change in CD163 or CCL18, the EV-MP group demonstrates that EVs provide both molecular cargo and biophysical stimuli needed for sustainable macrophage reprogramming. EVs function not merely as delivery vehicles but as architectural tools that modify macrophage shape, surface receptor engagement, and metabolic commitment—all critical determinants of M2 polarization [57,58].

In sum, these findings highlight the multifaceted immunomodulatory potential of fibroblast-derived extracellular vesicles (EVs) within an engineered 3D microenvironment. The concurrent elevation of metabolic activity and substantial upregulation of anti-inflammatory genes and surface proteins in the EV-MP group provides compelling evidence supporting the use of EVs in injectable biomaterial systems. Such integration holds promise for enhancing scaffold integration, regulating immune responses, and ultimately promoting tissue regeneration. However, the current study is not without limitations. A key limitation lies in the inability to directly correlate the observed macrophage responses with EV uptake. Both macrophages and EVs were co-encapsulated within a 3D polycaprolactone (PCL)-collagen matrix, which posed several technical challenges for real-time visualization of EV internalization. First, the matrix’s inherent opacity and substantial volume (~500 μL) hindered the acquisition of high-resolution, in situ confocal images. Attempts to liberate macrophages for ex vivo analysis of EV uptake also proved challenging. The enzymatic digestion required to extract intact cells from the composite matrix was complicated by the presence of PCL which necessitated higher concentrations of collagenase. This increased enzymatic strength risked compromising cell membrane integrity, thereby preventing reliable EVs uptake assessment.

Despite these limitations, the study provides strong fundamental evidence that fibroblast-derived EVs can modulate macrophage phenotype when both encapsulated together within 3D scaffold setting, offering a promising strategy for immuno-instructive tissue engineering platforms.

## 5. Conclusions

This study demonstrates the potential of integrating fibroblast-derived extracellular vesicles (EVs) into a three-dimensional (3D) injectable nanofibrous scaffold composed of polycaprolactone (PCL) nanofibers and collagen (PNCOL) to modulate macrophage behavior and preserve scaffold integrity under inflammatory conditions. EVs were isolated from human fibroblasts, characterized by size distribution and EV-specific surface markers, and incorporated into PNCOL scaffolds alongside pro-inflammatory (M1) macrophages and fibroblasts. Structural and immunological assessments revealed that EV-loaded scaffolds preserved matrix integrity, enhanced macrophage metabolic activity, and promoted an anti-inflammatory M2-like macrophage phenotype, as evidenced by reduced CD80 expression, increased CD206 expression, and upregulation of anti-inflammatory genes (TGF-β, CD163, CCL18). These findings highlight that fibroblast-derived EVs act as potent immunomodulatory agents capable of enhancing scaffold performance by simultaneously preserving matrix architecture and promoting anti-inflammatory macrophage responses.

## Figures and Tables

**Table 1:** Forward and reverse primers for real-time PCR.

Gene	Forward Primer 5′-3′	Reverse Primer 5′-3′	Ref.
CCL18	AAGAGCTCTGCTGCCTCGTCTA	CCCTCAGGCATTCAGCTTAC	[22]
TNF-α	AGAGGGAAGAGTTCCCCAGGGAC	TGAGTCGGTCACCCTTCTCCAG	[22]
IL-1β	AGCCATGGCAGAAGTACCTG	CCTGGAAGGAGCACTTCATCT	[22,34]
CD163	TCTGTTGGCCATTTTCGTCG	TGGTGGACTAAGTTCTCTCCTCTTGA	[22]
TGF-β1	GGTTATCTTTTGATGTCACCG	GTTATGCTGGTTGTACAGGG	[35]
GAPDH	AGAAGGCTGGGGCTCATTTG	AGGGGCCATCCACAGTCTTC	[22]

## Data Availability

The datasets generated during the current study are not publicly available due to the pending patent application but are available from the corresponding author on reasonable request.
